# The association of fasting blood glucose (FBG) and waist circumference in northern adults in Iran: a population based study

**DOI:** 10.1186/2251-6581-13-2

**Published:** 2014-01-06

**Authors:** Gholamreza Veghari, Mehdi Sedaghat, Hamidreza Joshaghani, Samieh Banihashem, Pooneh Moharloei, Abdolhamid Angizeh, Ebrahim Tazik, Abbas Moghaddami, Karimollah Hajian-Tilaki, Yedolla ZahedPasha

**Affiliations:** 1 Ischemic Disorders Research Center, Golestan University of Medical Sciences, Gorgan, Iran; 2 Deputy of Health; Golestan University of Medical Sciences, Gorgan, Iran; 3 Metabolic Disorders Research Center, Golestan University of Medical Sciences, Gorgan, Iran; 4 Department of Social Medicine and Health, Babol University of Medical Sciences, Babol, Iran; 5 Non-Communicable Pediatrics Disease Research Center, Babol University of Medical Sciences, Babol, Iran

**Keywords:** FBG level, Age, Adult, WC, Gender, Iran

## Abstract

**Objectives:**

The aim of this study was to evaluate the association between Fasting Blood Glucose (FBG) level and Waist Circumference (WC) in men and women among 25–65 years old people in the north of Iran.

**Material and methods:**

This was a cross-sectional and analytical research gender that carried out on the 1797 subjects (941 males and 856 females) between 25–65 years old using multistage cluster sampling technique. FBG was measured in the morning after a 12-hour fast and was determined by using laboratory kits (enzymatic methods) and spectrophotometry technique. Central obesity was defined based on World Health Organization criteria: waist circumference ≥102 cm and ≥88 cm in men and women, respectively. The SPSS.16 software was used for statistical analysis.

**Results:**

As whole, the mean of FBG in women (98.3 ± 40.1 mg/dl) was higher than in men (94.6 ± 32.2 mg/dl). Also, the mean of WC in men 4.5 cm was lower than in women. In men, the mean of FBG statistically differs between normal and central obese subjects both in 35–45 year-age group (P = 0.001) and in 45–55 year-age group (P = 0.042). As whole, in men, the FBG level increased up 2.82 mg/dl in each 10 cm of WC with the highest rate in 35–45 year-age group. In totally, in women, the FBG level increased up 3.48 mg/dl in each 10 cm of WC and in 25–35 year-age group and it was higher than in other age groups. In men, the regression coefficients were constant with age increasing while in women it was decreased. Constant trend in men and decreasing trend in women with age was shown between FBG and WC. The cut-off point of WC for detecting of diabetes obtained 89 cm and 107 cm in men and women, respectively.

**Conclusion:**

The positive correlation was seen between WC and FBG level and it was declined with age in women. Cut-off point for detecting of diabetes in men was less than in women. WC is useable as a predictor of type 2 diabetes mellitus risk among adults in the north of Iran.

## Introduction

Obesity, general and abdominal, is one of the major public health challenges for the current century with particularly alarming trends in several parts of the world. In 2005, the estimated total numbers of overweight and obese adults in worldwide, were 937 million and 396 million respectively, [1] numbers that have doubled in comparison to 20 years ago [2].

Waist circumference (WC) is an indicator to determine the central obesity [3,4] and it was considered as a risk factor for cardiovascular disease, stroke and type 2 diabetes [5,6]. It is well known that obesity, especially abdominal obesity, increases the risk of developing type 2 diabetes mellitus (T2DM) [7] and central obesity was significantly related to the plasma resistin levels [8]. The risk of T2DM in the obese can be described by changes in adipose tissue function [9-11]. The insulin resistance is associated with a higher plasma TG and lower HDL-C concentrations [12].

Studies have shown that the prevalence of abdominal obesity to range between 9.7 - 12.9% and 54.5 - 63.7% in Iranian men and women respectively [13,14]. Abdominal obesity investigated as a most health problem in the north of Iran [15,16].

Golestan province is located in the north of Iran (south east of Caspian Sea). Of 1,6 million populations in this area, 66.39% were 15–64 years old, whereas 43.9% and 56.1% were living in urban and rural areas, respectively. Agriculture is the main job in rural area and different ethnic groups such as Fars-native, Turkman and Sisstani are living in this region [17].

Since to our knowledge, no study has been conducted on the comparison of FBG between central obesity and normal subjects in the north of Iran, this study was designed and established in this area. The aim of this study was to evaluate the association between FBG level and WC in men and women among 25–65 years old people in the north Iran.

## Material and methods

This is a population based and cross-sectional study that conducted on the 1797 subjects aged 25–65 years (941 men and 856 women). The sample size estimated by previous study [18] and 95% confidence interval. The cases were chosen by stratified cluster sampling. From 11 districts, 100 clusters of 20 cases were randomly selected by family code in Primary Health Centers in rural areas and by zip code in urban areas with equal proportions of genders and age groups. From each district, one trained team to measured anthropometric indexes and recorded the demographic characteristics.

All family members in blocks who were in 25–65 years were included the clusters. The subjects had previously been identified as diabetic patient, pregnant women, diagnosed patients, on lowering drugs and those who were uninterested to participate in this study were excluded from the study.

Waist circumference was measured to the nearest 0.5 cm at the superior border of the iliac crest. Central obesity was defined after WHO criteria: waist circumference ≥102 cm and ≥88 cm in men and women, respectively [19]. For measuring of FBG, blood was drowned from each subject after 12 hours fast in the morning. FBG were determined using laboratory kits (enzymatic methods) and spectrophotometry technique. According to American Diabetes Association (ADA) criteria, FBG equal to or more than 126 mg/dl was diagnosed as type 2 DM [20].

SPSS 16.0 software was used for the statistical analysis and used Pearson correlation coefficients were calculated between WC and serum FBG and t.test to compare the means. We used linear regression model to estimate of regression coefficient of WC (in each 10 cm of WC) for prediction of FBG. The receiver operating characteristic (ROC) curves were employed to achieve the cut-off points of WC for detecting diabetes. The P. value under 0.05 included significations. This study was approved by the Ethical Research Committee and written informed consents were obtained from all the participants.

## Results

The mean of age in men and women was 44.3 ±11.4 and 44.1 ± 11.2 years, respectively. As whole, the mean of FBG in women (98.3 ± 40.1 mg/dl) was higher than in men (94.6 ± 32.2 mg/dl). Also, the mean of waist circumference in men 4.5 cm was lower than in women. Central obesity was common in 40.9% with a more prevalence in women (47.4%) than men (34.9%) and statistical differences was significant (P = 0.001).

The comparison of FBG between central obese and normal subjects based on age and sex was presented in Tables 1 and 2. In men, as whole the mean of FBG in central obese was 7.7 mg/dl more than normal people (P = 0.001). The mean of FBG statistically differs between normal and central obese subjects both in 35–45 year-age group (P = 0.001) and in 45–55 year-age group (P = 0.042). In totally, in men, the FBG increased up 2.82 mg/dl in each 10 cm of waist circumference and in 35–45 year-age group was higher than in other age groups (3.74 mg/dl). Also, in this age group was shown the highest positive correlation between FBG and WC compared with other age groups (r = 0.229, P = 0.001). There was a positive significant correlation between FBG and WC in whole of men subjects (r = 0.178, P = 0.001). Using ROC analysis, the cut-off point of WC according to maximum sum of sensitivity and specificity for detecting diabetes in men was 89 cm, while in aged 45–55 years it was 81 cm.

**Table 1:** The comparison of FBG level and WC based on age in men

**Age group (year)**	**No**	**WC status**	**FBG Mg/dl mean ± SD**	**P. value**	**Cut-off points of WC (cm)#**	**Regression coefficient (β)**	**Correlation coefficient (r)**	**P. value**
25–35	189	Normal	86.1 ± 20.5	0.451	90	0.196	0.138	0.029
	58	Central obese	88.2 ± 10.4					
35–45	160	Normal	86.5 ± 12.8	0.001	92	0.374	0.229	0.001
	81	Central obese	98.9 ± 29.9					
45–55	123	Normal	89.2 ± 17.3	0.042	81	0.218	0.139	0.035
	104	Central obese	94.7 ± 23.6					
55–65	141	Normal	91.0 ± 18.2	0.052	89	0.276	0.162	0.015
	85	Central obese	98.4 ± 31.9					
Total (941)	613	Normal	87.9 ± 17.6	0.001	89	0.282	0.178	0.001
	328	Central obese	95.6 ± 26.3					

*WC* waist circumference # Cut-off points of WC for detecting of diabetes.

**Table 2:** The comparison of FBG level and WC based on age in women

**Age group (year)**	**No**	**WC status**	**FBG Mg/dl mean ± SD**	**P. value**	**Cut-off points of WC (cm)#**	**Regression coefficient (β)**	**Correlation coefficient (r)**	**P. value**
25–35	164	Normal	84.1 ± 11.8	0.039	104	0.590	0.265	0.001
	61	Central obese	99.8 ± 55.4					
35–45	110	Normal	87.0 ± 15.4	0.043	110	0.373	0.216	0.001
	113	Central obese	94.3 ± 34.6					
45–55	82	Normal	87.5 ± 12.8	0.009	144	0.302	0.215	0.002
	128	Central obese	94.8 ± 22.7					
55–65	94	Normal	96.7 ± 27.5	0.774	64	0.051	0.030	0.676
	104	Central obese	95.7 ± 19.1					
Total (856)	450	Normal	88.1 ± 17.7	0.001	107	0.348	0.200	0.001
	406	Central obese	95.3 ± 32.3					

*WC* waist circumference # Cut-off points of WC for detecting of diabetes.

In women, the mean of FBG was 7.2 mg/dl in central obese more than in normal people (P = 0.001). In 55–65 year-age group, both the correlation between WC and FBG and mean differences of FBG between normal and obese subjects were not significant, while it was significant in other age groups (P < 0.05). In women, the FBG increased up 3.48 mg/dl in each 10 cm of waist circumference and it was higher in 25–35 year-age group than in other age groups (5.90 mg/dl). Also, compared with other age groups, in this age group the highest positive correlation was shown between FBG and WC in the 25–35 years age group (r = 0.265, P = 0.001). Using ROC analysis, the cut-off point of WC according to maximum sum of sensitivity and specificity for detecting diabetes in women was 107 cm, while in aged 55–65 years it was 64 cm.

**Figure 1:**
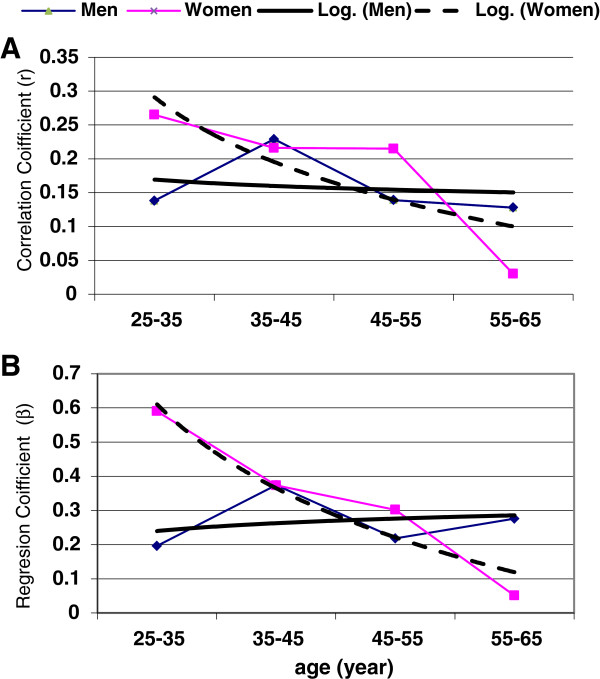
The comparison of genders based on (A) correlation coefficients changing and (B) mean FBG changing in per unit of WC among age groups.

The correlation corves between FBG and WC based on age groups and genders put on show in Figure 1. In men, the regression coefficients were constant with age increasing however among women this trend was declined. This change was shown in FBG in per unit WC increase.

## Discussion

Our finding shows that central obesity is a health problem in more than one third of the men and in half of the women among adults in the north of Iran. The positive correlation between FBG and WC was seen in both genders. The cut-off point of WC for detecting of diabetes in men is lower than in women.

The central obesity as a health problem reported in the north of Iran [21] and in whole of Iran [22] and most of diabetic patients in the north of Iran were unaware about their problem [23].

Present study revealed a positive correlation between FBG level and WC. Previous studies [24-26] were shown the similar results and Insulin resistant was seen among central obese subjects [27,28].

In our study, the correlation values between FBG and WC in all age groups were not equal while the strongest relationship was seen in men aged under 35 years and in women aged over 35 years. Similar study among Asian Indian adolescents reported that the prevalence of glucose intolerance is high, particularly in girls with abdominal obesity [29]. In similar reports [26,30] the optimal cut-off points of intolerant of T2DM risk was different in genders. In Bangladesh, [31] Impaired Glucose Tolerance and Impaired Fasting Glucose were more prevalent in female than in male and in Chennai Urban Population [32] the subjects belonging to higher socio-economic status had five times greater prevalence of glucose intolerance compared to subjects from lower socioeconomic. Dissimilation of association between WC and FBG in two genders may be due to physiologic mechanism.

In present study, the cut-off point of WC for detecting of diabetes was 89 cm and 107 cm in men and women, respectively. In a comprehensive study in Iran [33], cut-off point of WC reported 89.4 cm for men and 96.2 cm for women. In Isfahan (a province in center of Iran), cut-off WC obtained 80.7 cm for men and 84.7 cm for women [34]. The cut-off point of WC for detecting of diabetes was seen 85 cm in men and 77 cm in women in Korean adults [35] and it was seen 85 cm in both genders in Tunesian adults [36]. In Iran [33,34], like ours, the cut-off point of WC for detecting of diabetes in women was higher than in men; besides, the average of cut-off point in our study in men was equal and in women was higher than in the whole of Iran [33].

However, in our findings, the genders’ difference of cut-off-values that is more in women consisted with other studies in Iran [33,34] but it was not comparable with WHO criteria for detecting of central obesity. This difference has been observed by other researchers in Iran [37-39] who believed that WHO cut-off value for central obesity is not proper for Iranian population and recommended new criteria for classification of central obesity in Iran. In that way, we recommended evaluating these subjects in a comprehensive study in the north of Iran.

Compared with other regions [35,36], in our area, the cut-off values is high. We don’t know, why the cut-off point of WC for detecting of diabetes in our area results is higher than the others. All of confounder factors that correlated with WC and FBG such as physical activities, ethnicity and food behavior were not assessed in our study and may be these factors influence on the WC indices. There is necessary to evaluate these factors in an extensive study in future.

Similar to our results, the age was shown as an influence factor in plasma glucose level [32]. In a rural area in Bangladesh [31], waist circumference in glucose-intolerant subjects was more than in normal glucose-tolerant group. Also, glucose-intolerant was increased with age in this group. Age has a substantial influence on the association between BMI, waist circumference and insulin level in severely obese children [40].

In our result surprising, in women aged 55–65 years value 64, which is very low, has been observed. In addition, the other findings of this correspondence with low regression coefficients and low correlation coefficient were not almost significant. Our result indicated WC was not able to predict the FBG. Thus the optimal cut-off-value revealed in our results may not be an proper, because, basically we couldn’t predict it in this age group.

In present study was not clear why contrary to men, the correlation between FBG and WC decreased in women when age adjusted. A possible reason could be that their subjects have more health care or the physiologic factors effect on the insulin resistance. There is necessary a clinical trial design for study on these differences.

This study supports the evidence that WC is a predictor for T2DM. In severely obese children WC better than BMI was investigated as a predictor of fasting blood insulin [40] and in other studies the main predictor of diabetes were BMI and WC [41,42].

We did not assess the role of physical activities and ethnic differences on the relationship between FBG and WC and insulin level was not measured among subjects. In addition the statistical power will be increased if we hence the sample size. These are limiting factors for our study.

## Conclusion

The positive correlation was seen between WC and FBG among adult people in the north of Iran however this correlation was not similar in men and women when adjusted with age. The correlation trends are steady in men while in women descend with age. The cut-off point of WC for detecting of diabetes was 89 cm and 107 cm in men and women, respectively. WC is useable as a predictor of T2DM in northern adults in Iran.

## Competing interests

The authors declare that they have no competing interests.

## Authors’ contributions

The study was designed and implemented by MS,SB,PM,AA,ET and AM. The manuscript was prepared by GV, HJ, KH and YZ. All authors read and approved the final manuscript.
